# The Views and Experiences of Smokers Who Quit Smoking Unassisted. A Systematic Review of the Qualitative Evidence

**DOI:** 10.1371/journal.pone.0127144

**Published:** 2015-05-26

**Authors:** Andrea L. Smith, Stacy M. Carter, Sally M. Dunlop, Becky Freeman, Simon Chapman

**Affiliations:** 1 Centre for Values, Ethics and the Law in Medicine, School of Public Health, University of Sydney, Sydney, New South Wales, Australia; 2 School of Public Health, University of Sydney, Sydney, New South Wales, Australia; 3 Cancer Screening and Prevention, Cancer Institute NSW, Sydney, New South Wales, Australia; 4 Prevention Research Collaboration, School of Public Health, University of Sydney, Sydney, New South Wales, Australia; University of Edinburgh, UNITED KINGDOM

## Abstract

**Background:**

Unassisted cessation – quitting without pharmacological or professional support – is an enduring phenomenon. Unassisted cessation persists even in nations advanced in tobacco control where cessation assistance such as nicotine replacement therapy, the stop-smoking medications bupropion and varenicline, and behavioural assistance are readily available. We review the qualitative literature on the views and experiences of smokers who quit unassisted.

**Method:**

We systematically searched for peer-reviewed qualitative studies reporting on smokers who quit unassisted. We identified 11 studies and used a technique based on Thomas and Harden’s method of thematic synthesis to discern key themes relating to unassisted cessation, and to then group related themes into overarching concepts.

**Findings:**

The three concepts identified as important to smokers who quit unassisted were: motivation, willpower and commitment. Motivation, although widely reported, had only one clear meaning, that is ‘the reason for quitting’. Willpower was perceived to be a method of quitting, a strategy to counteract cravings or urges, or a personal quality or trait fundamental to quitting success. Commitment was equated to seriousness or resoluteness, was perceived as key to successful quitting, and was often used to distinguish earlier failed quit attempts from the final successful quit attempt. Commitment had different dimensions. It appeared that commitment could be tentative or provisional, and also cumulative, that is, commitment could be built upon as the quit attempt progressed.

**Conclusion:**

A better understanding of what motivation, willpower and commitment mean from the smoker’s perspective may provide new insights and direction for smoking cessation research and practice.

## Introduction

Research into smoking cessation has achieved much. Researchers have identified numerous variables related to smoking cessation and relapse, including heaviness-of-smoking, quitting history, quit intentions, quit attempts, use of assistance, socio-economic status, gender, age, and exposure to mass-reach interventions such as mass media campaigns, price increases or retail regulation.[1] Behavioural scientists have developed a range of health behaviour models and constructs relevant to smoking cessation, such as the theory of planned behaviour, social cognitive theory, the transtheoretical model and the health belief model.[2–5] These theories have provided constructs to smoking cessation research such as perceived behavioural control, subjective norms,[2] outcome expectations, self-regulation,[3] decisional balance,[4] perceived benefits, perceived barriers and self-efficacy.[5] The knowledge generated has informed the development of a range of pharmacological and behavioural smoking cessation interventions. Yet, although these interventions are efficacious,[6–8] the majority of smokers who quit successfully do so without using them, choosing instead to quit unassisted, that is without pharmacological or professional support.[9,10] Many smokers also appear to quit unplanned as a consequence of serendipitous events,[11] throwing into question the predictive validity of some of these cognitive models.

The enduring popularity of unassisted cessation persists even in nations advanced in tobacco control where cessation assistance such as nicotine replacement therapy (NRT) and the stop-smoking medications, bupropion and varenicline, are readily available and widely promoted.[9,10] Yet little appears to be known about this population or this self-guided route to cessation success. In contrast, the phenomenon of self-change (also known as natural recovery) is comparatively well documented in the fields of drug and alcohol addiction,[12,13] and health behaviour change (for example, eating disorders, obesity and gambling).[14]

We recently published a systematic review of unassisted cessation in Australia.[9] We, like others,[15] established that the majority of contemporary cessation research is quantitative and intervention focused.[16] While completing that review we determined that the available qualitative research was concerned primarily with evaluating smoker and ex-smoker perceptions of mass-reach interventions such as marketing or retail regulations, tax increases, graphic health warnings, smoke-free legislation or intervention acceptability from the perspective of the GP, current smoker, or third parties likely to be impacted by mass-reach interventions. Australian smoking cessation research provided few insights into quitting from the perspective of the smoker who quits unassisted. However our systematic review highlighted that 54% to 69% of ex-smokers quit unassisted and 41% to 58% of current smokers had attempted to quit unassisted.[9]

We consequently became interested in what the qualitative cessation literature had to say about smokers who quit unassisted. Qualitative approaches offer an opportunity to explain unexpected or anomalous findings from quantitative research and to clarify relationships identified in these studies.[17,18] By integrating individual qualitative research studies into a qualitative synthesis, new insights and understandings can be generated and a cumulative body of empirical work produced.[19] Such syntheses have proven useful to health policy and practice.[20,21] By focusing our review on the views of smokers (i.e. on the people to whom the interventions are directed), we might start to better understand why many smokers continue to quit unassisted instead of using the assistance available to them. Such an understanding might help us to decide whether we should be developing better approaches to unassisted cessation or focusing our attention on directing more smokers to use the efficacious pharmacological and professional behavioural support that already exists.

In this review, we examined the qualitative literature on smokers who quit unassisted in order to answer the following research questions: (1) How much and what kind of qualitative research has explored unassisted cessation? (2) What are the views and experiences of smokers who quit unassisted?

## Methods

Our qualitative synthesis is based largely on Thomas and Harden’s method of thematic synthesis.[20]

### Identification of articles for review

**Fig 1:**
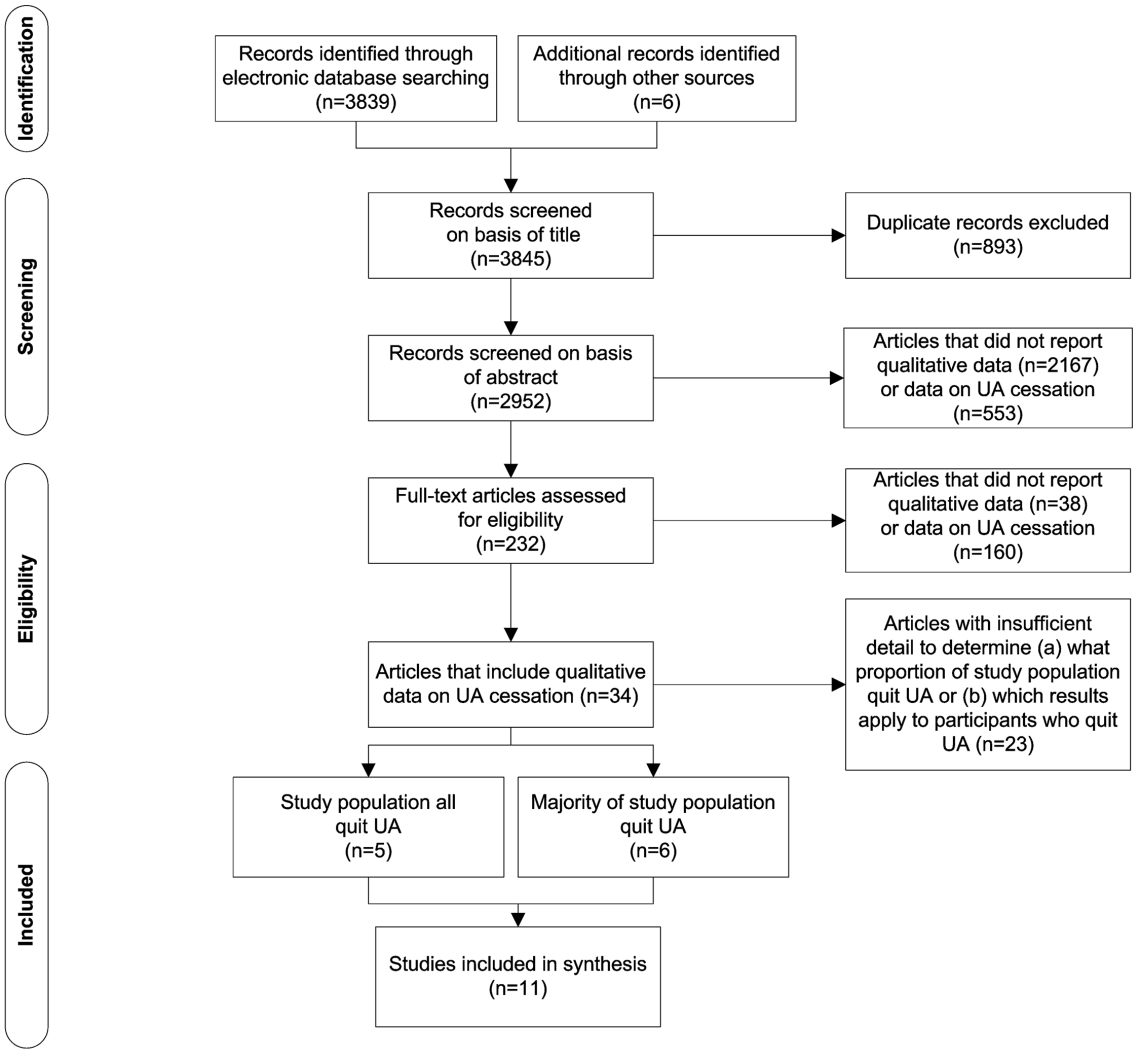
Identification and screening of eligible papers for inclusion in the synthesis.

We searched MEDLINE via OvidSP, PsycINFO via OvidSP, CINAHL via EBSCO, EMBASE and Sociological Abstracts in September 2013 for articles reporting on views about or experiences of quitting without assistance. Current best practice for identifying qualitative research recommends comprehensive searches of multiple sources, balancing sensitivity against specificity to maximise number of records retrieved while reducing retrieval of records that are not relevant.[22] We used empirically derived qualitative research filters where available (MEDLINE,[23] CINAHL[24] and PsycINFO[25]) (Table 1). We complemented this search strategy by conducting ‘berry picking’,[26] including grey literature searching, reference checking and author searching to uncover articles that are difficult to locate by modifying search terms and shifting searching strategies (Fig 1).

**Table 1:** Search terms.

Database	Search period^a^	Search strategy	Retrieved
MEDLINE via OvidSP	1946–Wk 3 Sept 2013	1. Smoking Cessation/	2318
		2. (interview: or experience:).mp. or qualitative.tw. or qualitative/	
		3. 1 and 2	
		4. remove duplicates from 3	
		5. limit 4 to (english language)	
PsycINFO via OvidSP	1806–Wk 3 Sept 2013	1. Smoking Cessation/	1058
		2. (experiences or interview: or qualitative).tw.	
		3. 1 and 2	
		4. remove duplicates from 3	
		5. limit 4 to (english language)	
EMBASE	1966–Wk 3 Sept 2013	1. ‘smoking cessation’/exp OR ‘smoking cessation program’/exp	225
		2. ‘qualitative research’/exp OR ‘qualitative research’	
		3. 1 and 2	
		4. 1 and 2 and [English]/lim	
CINAHL via EBSCO	1982–Wk 3 Sept 2013	1. ((ti interview or ab interview) or (mh "audiorecording" not mm "audiorecording") or (ti qualitative stud* or ab qualitative stud*))	176
		2. (MH "Smoking Cessation") OR (MH "Smoking Cessation Programs") OR (MH "Smoking Cessation Assistance (Iowa NIC)")	
		3. 1 and 2	
Sociological Abstracts	1952–Wk 3 Sept 2013	1. smok* and (quit* or cessation)	62
		2. qualitative	
		3. 1 and 2	
		**Total retrieved**	**3839**

^a^ All databases were searched on 24 September 2013.

Records were included if: (1) the article reported on the views or experiences of smokers or ex-smokers who quit; (2) the data collection and analysis methods were reported as qualitative by the authors; and (3) the article was in English. We set no date limits believing that early research was as likely to provide insightful data as more contemporary research and anticipating our search would produce relatively few studies. Screening was multi-levelled and moved from liberal to more specific. At the first level of screening (title and abstract), the focus was primarily on whether the study reported on unassisted cessation as abstracts often provided limited, incomplete or insufficient detail to make good decisions about inclusion based on methodological requirements.[27] We identified 3845 reports of which 11 met the inclusion criteria for the synthesis (Fig 1 and Table 2).

**Table 2:** Details of 11 studies included in the synthesis.

Source paper	Study year; country; setting	Study focus	Participants and participant characteristics	Participants who quit UA^a^	Data collection and analysis
Baer et al. 1977 [28]	Year not stated; US; community	Quitting without assistance	N = 51 (29 men, 20 women, 2 unknown; aged 29–75 years); ex-smokers who had smoked 2+ packs/day for 5+ years, who quit without professional direction or help, and had been an ex-smoker for 2+ years	All quit UA	Letters; convenience sample; content analysis
Solheim 1989 [29]	Year not stated; US; community	Quitting without assistance	N = 13 (7 men, 6 women; aged 25–49 years); ex-smokers who quit >6 months <2 years without assistance of a formalised intervention program; had previously smoked 0.5 pack/per day for >1 year prior to quitting	All quit UA	Semi-structured interviews; convenience sample; data analysis method not explicitly stated—included coding according to categories based on theoretical framework and interview guide
Thompson 1995 [30]	Year not stated; US; students and church groups	Successful cessation in women	N = 10 (all women; aged 28–48 years); women who had successfully quit smoking; smoked 10+CPD for 1+ year, and had quit >6 months but <3 years	8/10 (80%) quit UA (1 NRT gum; 1 hypnosis); 5/10 had previously used NRT patches unsuccessfully	Semi-structured interviews; purposive sample; data analysis based on Miles and Huberman 1994
Mariezcurrena 1996 [31]	Year not stated; Sweden; community	Recovery from addictions (tobacco, snus, drug, alcohol) without formal treatment	Total N = 58; ex-smokers = 38 (8 women, 30 men; aged 24–75 years); ex-smokers; ceased smoking 2+ years with no treatment or intervention (including prior treatment)	All quit UA	Semi-structured interviews; convenience sample; data analysis based on thematic analysis
Stewart 1999 [32]^b^	Year not stated; US; community	Spontaneous recovery from smoking	N = 40 (21 females, 19 males; aged 30–80 years); ex-smokers; tobacco free for 5+ years without the aid of any self-help or formal treatment programs	All quit UA	Semi-structured interviews (each participant was interviewed 2–4 times); convenience sample; data analysis used Spradley’s Development Research Model
Abdullah and Ho 2006 [33]	2002; Hong Kong; secondary school	Adolescents’ attitudes to smoking, quitting and smoking cessation programs	N = 32 (all male students in forms 2–4 equivalent to US grades 8–10); current smokers (n = 23) and ex-smokers (n = 9); 26/32 had attempted to quit, of whom 25/26 had attempted to quit UA	25/32 (78%) quit UA	5 focus groups; convenience sample; modified grounded theory
Nichter et al. 2007 [34]	2000–2002; US; Women, Infants & Children’s clinics, family practice offices, community	Factors contributing or undermining quit attempts/harm reduction at onset of pregnancy	N = 53 (all women); includes 2 case studies of women who quit UA or with minimal support (aged 20 and 31 years); low income women who were daily smokers at onset of pregnancy; (16 quit; 23 cut down; 14 continued smoking)	2 case studies of women who quit UA or with minimal support	Semi-structured interviews (each participant interviewed x3); convenience sample; discourse analysis.
Ogden and Hills 2008 [35]	Year not stated; UK; community	Mechanisms in sustained changes in behaviour (including those who lost weight through diet or exercise; and those who stopped smoking)	Total N = 34; ex-smokers = 10 (6 men, 4 women; aged 25–53 years); ex-smokers who had been quit for 3+ years; (8 quit UA; 2 with A—smoking course); quit 3–20 years ago	8/10 (80%) quit UA	Semi-structured interviews; convenience sample/possibly purposive; thematic analysis based on Huberman and Miles 1994
Bottorff et al. 2009 [36]	2006–2007; Canada; hospital antenatal units	How new fathers talk about the experience of tobacco reduction or cessation	N = 29 new fathers; ex-smokers who quit prior to birth of baby; (4/29 quit)	4/4 quit UA (remaining participants did not quit)	2x semi-structured interviews with each participant; convenience sample; narrative analysis
Murray et al. 2010 [37]^c^	2008; UK; general practice	The process of unplanned quit attempts and use of support in these attempts	N = 20 (11 male, 4 female); current and ex-smokers (15 ex-smokers, 5 current smokers); 7/10 spontaneous quitters quit UA; 1/10 delayed quitters quit UA	7/10 (70%) of spontaneous quitters quit UA	Semi-structured interviews; convenience sample; thematic analysis based on Ritchie 1994
Medbø et al. 2011 [38]	2008; Norway; community	Why older people smoke, why they quit and remain quit	N = 18 elderly persons (aged 58–80 years); ex-smokers (n = 13) and relapsed smokers (n = 5) all of whom had made temporary stops	“Majority of quitters had stopped themselves without medication”	Semi-structured interviews; convenience sample; content analysis (using a narrative perspective)

^a^ Studies were only included if the majority of participants quit unassisted (UA) and it was clear that the data analysis/findings were reporting on smokers who quit unassisted.

^b^ Stewart’s 1998 doctoral thesis,[39] on which this paper is based, was also checked for additional data.

^c^ Murray’s 2009 doctoral thesis,[40] on which this paper is based, was also checked for additional data.

### Quality appraisal

We are aware that structured approaches to quality appraisal (such as guidelines and checklists) do not necessarily produce greater consistency of judgements about which papers to include in a qualitative synthesis.[41] Concern with procedural correctness can unduly focus attention on the reporting of the research process and divert attention away from the analytical content of the research.[42] Authors of previous qualitative syntheses have reported a disconnect between papers they believed intuitively to be well conducted research and those that ‘passed’ when assessed against structured quality assessment criteria.[18] We knew that many of the articles identified in our searches would have been published prior to the development of quality appraisal checklists. The retrospective application of a tool developed many years after a study’s publication appeared inappropriate given the changing norms around the reporting of qualitative research. We decided, like Thomas and Harden[20] and others (e.g., Atkins 2008[18] and Lipworth 2011[43]), to err on the side of inclusion and to judge quality on the basis of conceptual contribution as much as methodological rigor.

### Extracting data

Our research questions were deliberately broad: (1) How much and what kind of qualitative research has explored unassisted cessation? (2) What are the views and experiences of smokers who quit unassisted? We were interested not only in what the data had to say about smokers who quit unassisted but also in gaining an understanding of the breadth of themes related to unassisted cessation. We treated as data anything reported in the results or findings sections (usually key concepts or findings, but also direct quotations) and, if relevant, the researchers’ interpretations of smokers’ and ex-smokers’ views and experiences, as reported in the discussion or conclusion of the article.

### Thematic synthesis

Data analysis involved three overlapping stages: (1) line-by-line coding of the results from the 11 primary studies followed by (2) grouping of the line-by-line codes into descriptive themes that related to (3) broader, overarching concepts.

During the initial line-by-line coding we read and closely examined fragments of data (words, lines, segments and incidents) for their analytical importance. Line-by-line codes were created to reflect what was happening in these ‘meaning units’, and to show actions; for example, what the participants were thinking, feeling or doing.[44] Next, the line-by-line codes that were conceptually similar were grouped into descriptive themes and then these descriptive themes were grouped into overarching concepts. Once all of the descriptive themes had been sorted and grouped into concepts, the analysis became more focused. We report here only on the most significant themes and concepts: those which were most original and about which the literature had most to say.

### Input from team members

The first author coded the primary studies and developed the descriptive themes and concepts. These were then discussed with the team as a whole and with team members individually. The team members brought to the analysis a range of professional experiences and perspectives relevant to smoking cessation (including qualitative health research, tobacco control and health behaviour change).

### Ethics statement

As this was a systematic review of existing studies no ethics approval was required.

## Results

Our findings are reported in two parts: (1) how much and what kind of qualitative research has explored unassisted cessation (Tables 3 and 4); (2) what are the views and experiences of smokers who quit unassisted? (Table 5).

**Table 3:** Overview of 11 studies synthesised.

	1970s	1980s–1990s	2000 onwards
**Number of studies**	1 [28]	4 [29–32]	6 [33–38]
**Disciplines**	Psychiatry	Sociology, nursing	Medicine, psychology, nursing, public health, community medicine
**Population**	General, mainstream	General, mainstream	Specific populations (e.g adolescents, the elderly, new parents)^b^
**Smoking and quitting status**	Ex-smokers, all unassisted	Ex-smokers; all unassisted^a^	Smokers, ex-smokers, relapsed smokers
**Primary focus**	Unassisted cessation	Unassisted cessation	Cessation in general, health behaviour change in general
**Aims**	Inform psychotherapy intervention design	Understand the phenomenon of unassisted cessation	Understand attitudes to cessation, reasons for quitting, reasons for relapse; inform intervention design

^a^ Thompson 1995 included 1 smoker who used NRT gum and 1 who used hypnosis to quit (out of a total of 10 ex-smokers); the focus was cessation in general rather than unassisted cessation in particular but as most participants quit unassisted the data were included in this synthesis.

^b^ Ogden and Hills 2008 and Murray 2010 reported on general rather than specific populations.

**Table 4:** Main themes and conclusions relating to UA quitting in the 11 studies and their contribution to the themes and concepts reported in this review.

Source paper	Main themes relating to UA quitting reported in this paper	Main conclusions relating to UA quitting reported in this paper	Concepts and themes reported in this review ^a^	Conceptual contribution to this review ^b^
Baer et al. 1977 [28]	(1) Pronounced differences in the techniques used by participants to quit; (2) Challenge to self and motivation appeared as a common combination of techniques, as did motivation and self-derogation	Most respondents used multiple techniques to quit, but there was no systematic clustering of these methods	Motivation—equivalent to one’s reason for quitting; Willpower—tautologous, ambiguous; Willpower—a personal quality or trait; Commitment—being serious or resolute	Medium
Solheim 1989 [29]	(1) Socio-environmental factors affect cessation (e.g. interactions with family, friends and health professionals); (2) Thoughts pre-quitting primarily negative (e.g. assessing benefits and consequences of smoking, or process of quitting). Thoughts post-quitting primarily positive; (3) Emotions pre-quitting included guilt, fear, anger, and disquiet. Emotions post-quitting are positive, but also included loss and resentment; (4) Motivational response included decision-making, self-determinism, taking action, messages to oneself	Smoking cessation is a process that begins before an individual stops smoking; characteristic thought processes and emotions occur before and after cessation; actions to aid cessation are unique to each individual; family and friends are influential; factors may be interactive, occur simultaneously and may be cumulative in their effect on the cessation process	Motivation—equivalent to one’s reason for quitting	Low
Thompson 1995 [30]	(1) Evolving commitment to health and personal growth; (2) The effect of a smoke-free environment; (3) The impact of anti-smoking education; (4) Changing conceptualisation of smoking	Anti-smoking education, coupled with smoke-free environment, augments the awareness of the effects of smoking and directly impacts on one’s conceptualisation of smoking	Willpower—a strategy; Commitment—being serious or resolute; Commitment— can be cumulative	Medium
Mariezcurrena 1996 [31]	(1) Triggers precipitating or helping quitting; (2) Coping strategies used to quit; (3) Advice given by ex-smokers about quitting (successful quitting required decision-making, wanting to stop, being determined, and belief in oneself)	Participants attributed their change to their own effort; making the decision to stop was the most frequent trigger to stopping; it was related to fear, health concerns and feeling of loss of control	Motivation—equivalent to one’s reason for quitting; Commitment—being serious or resolute	Low
Stewart 1999 [32]	(1) Contemplation: allows for goal setting; mental preparation; knowledge of addiction; (2) Decision to quit: unique decision; allows for no excuses; willpower; no desire to smoke; (3) Relapse: creates knowledge of pitfalls; less commitment in previous attempt; life events cannot overwhelm willpower; no moderation; (4) Environment: contributed to smoking; motivation; attitude towards other smokers; (5) Process of cessation: multiple techniques; point of no return; dreams	Participants used multiple techniques to quit; most had relapsed and used this as motivation to continue trying to quit	Motivation—equivalent to one’s reasons for quitting; Motivation—not a prerequisite for quitting; Willpower—tautologous, ambiguous; Willpower—a method; Willpower—a personal quality or trait; Commitment— being serious or resolute; Commitment—can be tentative or provisional; Commitment—can be cumulative	High
Abdullah and Ho 2006 [33]	Themes (importance of quitting, perceived barriers to quitting, perceived benefits of quitting, reasons to quit) were general and reported little specifically about UA quitting	Decision to quit smoking was not an urgent or important decision; belief that they could quit at any time with little difficulty; willpower and determination can help quitting	Willpower—tautologous, ambiguous; Willpower—a personal quality or trait	Low
Nichter et al. 2007 [34]	(1) Reasons for quitting (for the baby, social pressure, fear, appeasing family); (2) Moral authority to control environments in which smoking is normative; (3) Smoking is a personal responsibility and quitting is a matter of personal choice	Successful quitters had a strong sense of moral identity as a mother; concern for effect of smoking on foetus; social networks had an important impact on woman’s ability to quit; lack of control of environment affected quitting success	Commitment—being serious or resolute	Low
Ogden and Hills 2008 [35]	(1) The role of life crises as specific triggers to initial behaviour change; (2) Key sustaining conditions (a disruption of function; a reduction in choice; behavioural model of causes and solutions) which allowed the initial change in behaviour to be translated into a longer term change in lifestyle	If a person no longer benefits from the behaviour, finds that they are fewer opportunities to carry out the unhealthy behaviour and believes that the behaviour was the cause of his or her problems, then an initial change in behaviour is more likely to be translated into a behaviour change in the longer term; central to all themes was a process of reinvention and a shift toward a new healthier individual	Willpower— a method	Low
Bottorff et al. 2009 [36]	(1) Cold turkey storyline framed quitting smoking as a snap decision with no need for support; (2) The ‘baby as the patch’ storyline dramatised how the baby displaced the need to smoke, increased motivation for cessation and enhanced success	Common to all storylines was the men’s reluctance to rely on smoking cessation resources; instead self-reliance, willpower, autonomy figured more prominently in the narratives	Motivation—equivalent to one’s reasons for quitting; Willpower—tautologous, ambiguous; Willpower—a personal quality or trait	Medium
Murray et al. 2010 [37]	(1) The majority of spontaneous quitters had not used any support; (2) Reasons for not using support included lack of time to access support, lack of knowledge about support available, belief general practitioner would not be receptive to offering smoking cessation support, a belief they should quit on own	The majority of spontaneous quit attempts were made without the use of support	Commitment—being serious or resolute	Low
Medbø et al. 2011 [38]	(1) Approaching a decision to stop: reflection on the consequences of smoking; ambivalence hardens into resolution and the smoker waited for an appropriate opportunity to quit; (2) The actual stopping: many stopped suddenly and unplanned as a result of accidental circumstances; no clear decision-making, stopping without visible internal struggle or resolution; (3) Quitting was easier than expected	Patient preferences for quitting should be explored; some smokers may stop unplanned with little motivation; GPs interest in the smoking narrative may sometimes be enough to encourage cessation	Motivation—not a prerequisite for quitting; Commitment—can be tentative or provisional	Low

^a^ Includes only the themes and concepts reported in this review, not all of the themes and concepts that were coded and mapped (see Fig 2 for full range of concepts).

^b^ Conceptual contribution to review: low: contributed to <3 themes; medium: contributed to ≥3–5 themes; high: contributed to ≥6 themes (see Table 5 for more detail on how individual studies contributed conceptually to the review).

**Table 5:** Concepts and descriptive themes derived from the 11 studies, with illustrative quotes.

Concepts	Descriptive themes	Reported in	Illustrative quotes^a^
Motivation	Equivalent to one’s reasons for quitting	Baer; Bottorff; Mariezcurrena; Solheim; Stewart	‘I got to thinking about how much money I spend on the habit’ *Informant quote in Baer;* ‘Guilt was experienced in relation to children’s health’ *Solheim;* ‘Motivational responses are derived from the individual’s need to feel competent and self-determining about his or her life, and stopping the habit of smoking meets this need’ *Solheim;* ‘I didn’t like the fact that cigarettes had so much control over where I went and what I did and who I went with … I wanted to be in control of my life*’ Informant 7 in Stewart*
	Not a prerequisite for quitting	Medbø; Stewart	‘Our findings indicate that it is possible to stop smoking even at very low levels of motivation’ *Medbø*
Willpower	Tautologous, ambiguous	Abdullah; Baer; Bottorff; Stewart	‘One is successful if one has willpower, one has willpower if one successfully quits’ *Stewart;* ‘Willpower is the answer’ *Baer*
	A method	Ogden; Stewart	‘The smoking group had stopped smoking either through will power or a smoking course’ *Ogden*
	A strategy	Thompson	‘Six of the women in the study used sheer will-power to overcome the strong urges to smoke they experienced’ *Thompson*
	A personal quality or trait	Abdullah; Baer; Bottorff; Stewart	‘Common to all the storylines was the men’s reluctance to rely on smoking cessation resources; instead self-reliance, willpower and autonomy figured more prominently’ *Bottorff*
Commitment	Being serious or resolute	Baer; Mariezcurrena; Murray; Nichter; Stewart; Thompson	‘One of the factors that did seem to differentiate this decision was that it was a firm decision. It was often described as the firmest commitment they had ever made.’ *Stewart;* ‘I was thinking too that before I actually quit, that the times before, subconsciously, I really didn't want to, or I wasn't taking the task seriously enough’ *Informant 4 in Stewart*
	Can be tentative or provisional	Medbø; Stewart	‘I always felt like it would be … OK, I’m going to give this a valiant attempt and if it’s not going to work, then I’ll go back to smoking and it will be OK’ *Informant 12 in Stewart;* ‘I had been working on my decision, you can say. I did not dread the stop because if it turned out to be too hard I would start smoking again’ *Informant quote in Medbø;* ‘I don’t think that in previous attempts that I ever decided that I would quit because I wanted to. I guess I never really wanted to stop’ *Informant 16 in Stewart*
	Can be cumulative (commitment builds as the quit attempt progresses)	Stewart; Thompson	‘You can’t quit [relapse] now you only have a little bit left’ *Informant 17 in Stewart;* ‘‘I knew that if I stopped [relapsed] it would have killed me. I had put too much time into this’ *Informant 38 in Stewart;* ‘The evolving commitment was also evident in words that echoed repeatedly a personal determination and desire to achieve the goal of quitting smoking. Declarations such as "I knew I could not turn back once I made my mind up"‘ *Thompson*; “It gets to the point where you know you can do it. You’ve got so much invested that if you [relapsed] it’d be really hard. At that point you just can’t [relapse]” *Informant 17 in Stewart*

^a^ The majority of the quotes report the study authors’ conclusions; the remainder are direct quotes from participants.

### Research question 1: How much and what kind of qualitative research has explored unassisted cessation?

The earliest study identified was a 1977 US study investigating why smokers seeking treatment (psychotherapy) often fared no better than smokers who quit unassisted.[28] This was followed in the late 1980s and 1990s by three in-depth sociological studies (from the US and Sweden) investigating unassisted cessation as a phenomenon in its own right,[29,31,32] and one US sociological study in which unassisted cessation data were reported but this was not the primary focus of the study.[30] Subsequent to this, no qualitative studies were identified that focused on unassisted cessation *per se*: the six post-2000 studies (from Hong Kong, US, UK, Canada and Norway) had as their primary focus either cessation in general[33,34,36–38] or health behaviour change.[35]

### Research question 2: What are the views and experiences of smokers who quit unassisted?

**Fig 2:**
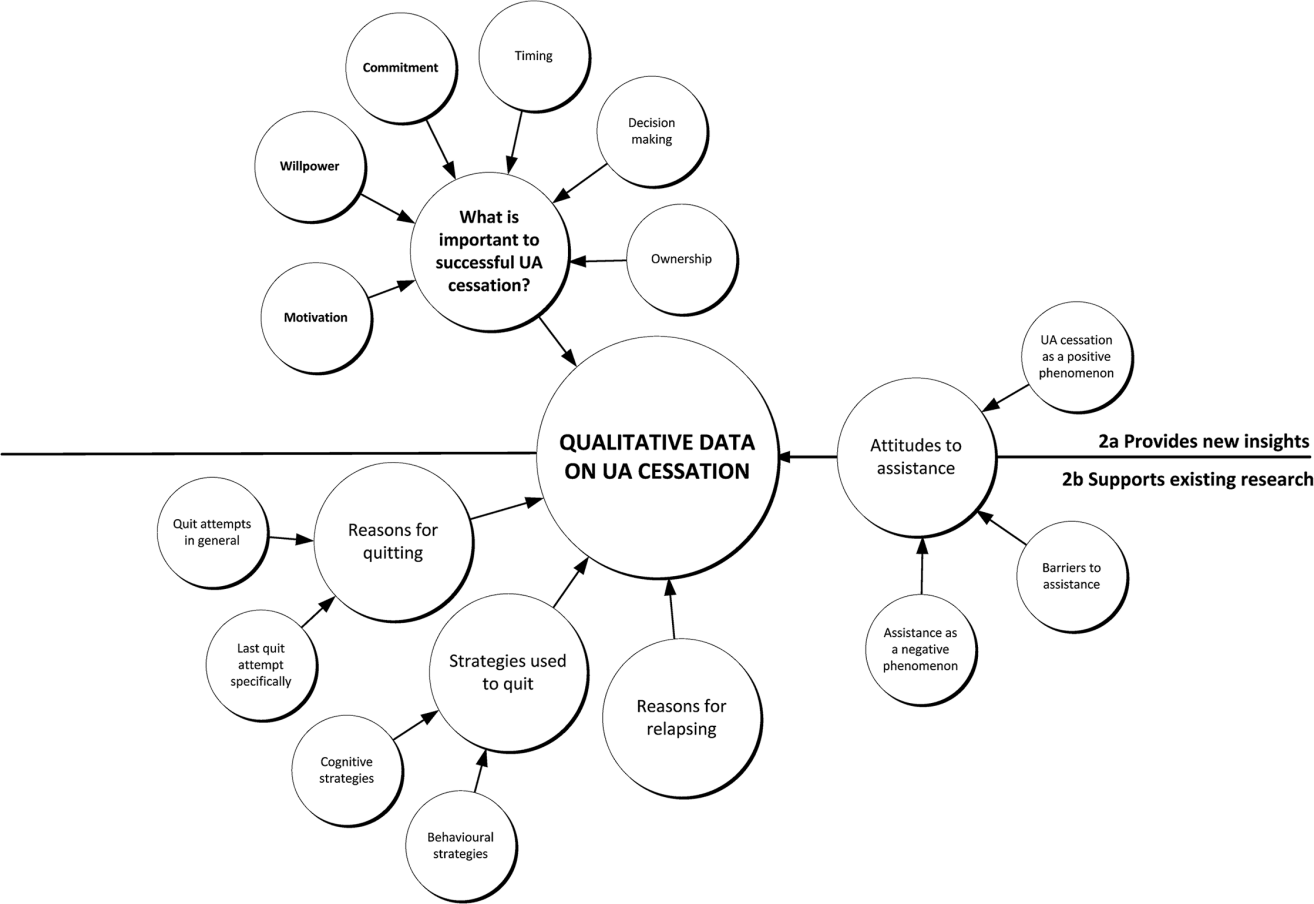
Themes and concepts derived from the 11 primary studies. UA, unassisted.

The full set of concepts derived from the qualitative literature is shown in Fig 2. Concepts were grouped into those that included descriptive themes that have already been covered in the literature (Fig 2B, below the line) and those concepts that included descriptive themes that provided potentially new insights into unassisted cessation (Fig 2A, above the line). The existing quantitative smoking cessation literature has, for example, already reported on attitudes to assistance, reasons for quitting, strategies used to quit and reasons for relapsing (Fig 2B). While encouraged by the consistency between the qualitative and quantitative studies, our aim was to focus on what the qualitative literature could report from the smokers’ perspective about quitting unassisted that had the potential to offer new or alternative insights into the process or experience of unassisted cessation (Fig 2A). From this perspective the most interesting themes were those that related to three concepts: (1) willpower; (2) motivation; and (3) commitment. Four further concepts (timing, decision-making, ownership and the perception that quitting unassisted was a positive phenomenon) were of interest but insufficient data were available on which to base an analysis.

We detail the three concepts that appeared central to smokers who self-quit (motivation, willpower and commitment) in Table 5. Although these concepts appear in the scientific and lay literature on smoking cessation, in the following section, we explore the meaning of these concepts as defined by smokers and ex-smokers who have quit unassisted, as well as the researchers who studied them.

#### Motivation

Although motivation was widely reported it was difficult to discern exactly what motivation meant to the smokers as opposed to the researchers. Smokers rarely talked directly about motivation or used the word motivation to describe their quit attempt. Yet motivation was frequently included in the accounts *researchers* gave of how and why smokers quit. That is, there appeared to be a disjunct between the way that researchers talked about motivation and the way that ex-smokers understood it. On looking at the data related to motivation it became clear that when researchers talked about motivation they were in fact talking almost exclusively about reasons for quitting. Typical reasons included cost,[28] a sense of duty,[28,29,31,32,36] health concerns,[28,29,31,32] feeling out of control, feeling diminished by being a smoker,[28,29,31,32] deciding the disadvantages of smoking outweighed the benefits,[29] or expectations that life would be better once quit.[29] We concluded the data on motivation reported in these 11 qualitative studies added no new insights to the data on reasons for quitting already reported in the quantitative literature.

Smokers used the word motivation differently: not to describe the reason they quit, but to describe what sustained them through their quit attempt. We have included these data under the concept of commitment (see below). Our main conclusion about motivation is that smokers and researchers appear to be using the word to denote different concepts.

#### Willpower

The concept of willpower was clearly important to smokers and often used by researchers to account for smokers’ success or failure, but rarely examined or unpacked. Willpower was reported to be a method of quitting, a strategy to counteract cravings or urges (much as NRT or counselling is regarded as a method of quitting or a way of dealing with an urge to smoke)[30,32,35] or a personal quality or trait fundamental to quitting success.[28,32,33,36] For example, although Ogden and Hill (2008) classified their participants according to whether they had ‘stopped smoking through willpower or a smoking course’, they gave no definition or explanation of what willpower was. Similarly, Thompson (1995) reported many participants used ‘sheer willpower to overcome the strong urges to smoke’, and Abdullah and Ho (2006) reported relapsed smokers cited ‘willpower and determination’ as key factors for quitting success, but did not elaborate on what was meant by willpower. Stewart’s 1999 sociological study of smokers who quit unassisted[32] attempted to understand willpower from the smokers’ perspective, yet despite directly questioning smokers about willpower, Stewart could find no agreement among smokers as to what willpower was. In summing up, Stewart concluded: ‘it is difficult to connect a successful cessation attempt with the use of willpower without creating a tautology: one is successful if one has willpower, and one has willpower if one is successful,’ capturing what is arguably still an issue in contemporary smoking cessation research.

#### Commitment

Smokers’ talk about commitment was nuanced and multilayered. In contrast to motivation and willpower we did not need to rely upon the researchers’ interpretations to gain an insight into what commitment might mean to smokers. Smokers talked directly about being committed. To them it meant being determined, serious or resolute. Being committed was essential to their quitting success.[28,30–32,34,37] Commitment was what differentiated a serious quit attempt from previous unsuccessful quit attempts,[32] and was the hallmark of their final successful quit attempt.

Commitment could also be tentative or provisional.[32,38] Medbø (2011) reported smokers who appeared keen to try to quit but were not necessarily committed to seeing the quit attempt through. It is possible a further level of commitment was being withheld, contingent perhaps on how difficult quitting turned out to be or on how the smoker felt about being quit once they got there. One of Stewart’s participants illustrates the difference, ‘OK I’m going to give this a valiant attempt and if it’s not going to work then I’ll go back to smoking and it will be OK.’[32] The smoker is committed to trying but not necessary committed to quitting.

Commitment could also be cumulative. Smokers talked about a point of no return, which described a point in the cessation process when they had made a firm commitment to quit, they had made a decision and they would not change their mind.[30,32] Smokers described this as the point in time at which they believed there was too much invested to relapse now.[32]

## Discussion

In this review we have synthesised the qualitative data reporting on the views and experiences of smokers who successfully quit unassisted (without pharmacological or professional behavioural support). The existence of only a handful of studies over more than 50 years, with no study specifically addressing unassisted cessation post-2000, indicates that up until now little research attention has been given to the lived experiences and understandings of smokers who successfully quit unassisted. As a consequence relatively little is known about smokers’ perspectives on what is the most frequently used means of quitting[10] and the way described by the majority of ex-smokers as being the most ‘helpful’.[45,46] It is widely accepted that searching the qualitative literature is difficult.[21,47] Although it is possible that relevant studies were missed, given the comprehensiveness of our search strategy, the comparative lack of studies found through searching seems likely to reflect an evidence gap, and therefore an important area for future research.

This lack of qualitative research was unexpected for two reasons. First, we were aware of a small but not unsubstantial body of quantitative evidence on smokers who quit unassisted;[48–52] and second, in the course of our literature search we had identified a considerable number of qualitative studies on smoking cessation. On closer examination it became clear that few of these reported specifically on smokers who quit unassisted. This supports what Kluge found in 2009, that is, the qualitative smoking cessation research that does exist is concerned primarily with evaluating the success or acceptability of smoking cessation interventions, particularly in vulnerable populations such as adolescents or the socially disadvantaged.[16]

### Concepts central to self-quitting

Motivation was identified as a central concept in this review, but analysis of the studies showed that motivation appeared primarily in the researchers’ accounts of quitting rather than in the smokers’ accounts of quitting. On closer examination, the data related to motivation consisted almost entirely of reasons for quitting. Within the quantitative literature on smoking cessation, motivation is an established psychological construct which has been operationalised in numerous studies designed to determine the role of motivation in quitting success.[53,54] Motivation has been identified as critical to explaining cessation success.[55] The lack of explicit discussion about motivation by smokers who quit unassisted in the studies included in this review is therefore interesting. Though motivation could be inferred from the smokers’ accounts; it had to be done by using the variables that comprise motivation, such as reasons (motives) for quitting or the pros and cons of smoking versus quitting. Given the relative lack of data, it is difficult to conclude whether this is (1) because smokers do not talk directly about motivation, or (2) whether from the participants’ perspective motivation is not the driving force behind successful unassisted cessation (either because another concept is more important or because too much time has passed since their quit attempt for them and they have forgotten how important motivation was to them).

From the studies included in this review, it appears that—at least in smokers’ self-understanding—commitment might be more important than motivation as an explanation of successful unassisted cessation. The enthusiastic and explicit talk about being determined, committed, or serious suggests that this concept resonates more with smokers than the concept of motivation. The overlapping and at times contradictory natures of commitment and motivation have been highlighted recently by Balmford and Borland who concluded that it may be possible to quit successfully while ambivalent, as long as the smoker remains committed in the face of ebbs and flows in motivation.[56] Further complicating the relationship, some regard commitment as a component of motivation,[57] operationalizing motivation as, for example, ‘determination to quit’[58] or ‘commitment to quit’.[59]

The greater research interest in reasons for quitting or pros and cons of quitting (i.e., motivation) as opposed to commitment may be because motivation is simpler to measure, for example by asking people to rate or rank reasons, costs or benefits. From a policy and practice perspective, it may also be easier to draw attention to these reasons, costs and benefits, rather than engage with commitment. For example, mass media campaigns can remind smokers of why they should quit by pointing out the benefits to short-term and long-term health. However this review draws attention to the importance of commitment for sustained quitting, at least from the point of view of smokers and quitters. The UK’s annual Stoptober campaign in which smokers committed to being smoke-free for 28 days indicates that creative approaches to addressing commitment can be successful.[60]

The final concept identified, willpower, was described in terms of multiple constructs (a personal quality or trait, a method of quitting, a strategy to counteract cravings or urges), suggesting smokers and researchers may use it as a convenient or shorthand heuristic when talking about or reporting on quit success. Despite this lack of clarity, the word has persisted in the qualitative and quantitative smoking cessation literature. It could be fruitful for future research to further examine the meaning of willpower, and particularly its relationship to other more tightly defined concepts such as self-efficacy,[61] self-regulation[62] and self-determination,[63] from the perspective of both researchers and smokers.

No matter how widely available and affordable smoking cessation assistance becomes, it is likely there will always be a significant proportion of smokers who choose to quit unassisted.[9] It is important to understand what drives these smokers to quit this way and to better understand their route to success. Orford and colleagues working on the UK Alcohol Treatment Trial made a strong case for including the client’s perspective, arguing that it is wrong to assume that clients have no perspective into their own change processes, and that we should resist the dominant ‘drug metaphor’ which has adopted the model of an active professional applying a technique to a passive recipient.[64] McDermott and Graham also advocated for the need for contemporary public health policy to ground itself in the experiences of those whose lifestyles it seeks to change.[65] As the vast majority of smokers who quit successfully continue to do so without formal help, it is likely that a better understanding of this experience, from the perspective of the smokers and ex-smokers themselves, could inform more nuanced and effective communication and support for quitting.

## Limitations

A potential limitation was the quality of the original articles. Our *a priori* decision not to assess articles based on the overall quality of the studies using standard guidelines or checklists meant that some of the included studies failed to report, for example, the theoretical framework, the sampling strategy, the procedures for data analysis, or the theoretical justification for their data analysis. In addition, several of the studies, especially those post-2000 reported data that were descriptive rather than analytic.

It is possible that we, the authors of the review, were sensitised to some of the themes identified as important due to our involvement in an ongoing grounded theory study into how and why smokers quit unassisted. Some of our interviews with ex-smokers took place at the same time as this qualitative review. The influence of this overlap, however, is likely to be minimised by the fact that not all authors had been involved in the data collection or analysis of the grounded theory study at the time of the review, and the whole authorship team were involved in the development and refinement of the analysis.

And finally, the three concepts identified are not discrete, and are likely to overlap in many ways. The studies identified confirmed the importance of these concepts, but did not always analyse them in sufficient detail to allow us to draw firm and transferable conclusions about their meaning. We have identified the importance of these concepts: further research is needed to strengthen our understanding of how smokers understand and use them.

## Conclusion

Our review identifies three key concepts, motivation, willpower and commitment, circulating in smokers’ and ex-smokers’ accounts of quitting unassisted. Insufficient qualitative evidence currently exists to fully understand these concepts, but they do appear to be important in smokers’ and ex-smokers’ accounts and so worthy of research attention. A more detailed qualitative investigation of what motivation, willpower and commitment mean to smokers and ex-smokers would complement the existing body of behavioural science knowledge in tobacco control. A better understanding of these concepts from the smokers’ perspective may help to explain the often-puzzling popularity of quitting unassisted rather than opting to use the efficacious pharmacological or professional assistance that is available. Health practitioners could potentially use such knowledge, in combination with what we already know from population-based research into smoking cessation, to better support all smokers to quit, whether or not they wish to use assistance.

## Supporting Information

S1 PRISMA Checklist PRISMA checklist (DOC)

## References

[1] Scollo MM, Winstanley MH (2012) Tobacco in Australia: Facts and Issues. Melbourne: Cancer Council Victoria.

[2] Ajzen I (1991) The theory of planned behavior. Organizational Behavior and Human Decision Processes 50: 179–211.

[3] Bandura A (1986) Social foundations of thought and action: a social-cognitive theory Englewood Cliffs, New Jersey: Prentice Hall.

[4] Prochaska JO, DiClemente CC (1983) Stages and processes of self-change of smoking: toward an integrative model of change. J Consult Clin Psychol 51: 390 6863699 10.1037//0022-006x.51.3.390

[5] Becker MH (1974) The health belief model and personal health behaviour Thorofare, New Jersey: Charles B Slack.

[6] Cahill K, Stevens S, Lancaster T (2014) Pharmacological treatments for smoking cessation. JAMA 311: 193–194. 10.1001/jama.2013.283787 24399558

[7] Stead LF, Hartmann-Boyce J, Perera R, Lancaster T (2013) Telephone counselling for smoking cessation. Cochrane Database of Systematic Reviews Issue 8 CD002850. 10.1002/14651858.CD002850.pub3 23934971

[8] Stead LF, Buitrago D, Preciado N, Sanchez G, Hartmann-Boyce J, Lancaster T (2013) Physician advice for smoking cessation. Cochrane Database of Systematic Reviews Issue 5 CD000165. 10.1002/14651858.CD000165.pub4 PMC7064045 23728631

[9] Smith AL, Chapman S, Dunlop SM (2013) What do we know about unassisted smoking cessation in Australia? A systematic review, 2005–2012. Tob Control tobaccocontrol-2013-051019. 10.1136/tobaccocontrol-2013-051019 24026163

[10] Edwards SA, Bondy SJ, Callaghan RC, Mann RE (2014) Prevalence of unassisted quit attempts in population-based studies: a systematic review of the literature. Addictive Behaviors 39: 512–519. 10.1016/j.addbeh.2013.10.036 24333037

[11] West R, Sohal T (2006) “Catastrophic” pathways to smoking cessation: findings from national survey. BMJ 332: 458–460. 16443610 10.1136/bmj.38723.573866.AE PMC1382540

[12] Slutske WS (2010) Why is natural recovery so common for addictive disorders? Addiction 105: 1520–1521. 10.1111/j.1360-0443.2010.03035.x 20707774

[13] Miller PM, Smith JP (2010) What do marshmallows and golf tell us about natural recovery research? Addiction 105: 1521–1522. 10.1111/j.1360-0443.2010.03034.x 20707775

[14] Sobell LC (2010) The phenomenon of self-change: overview and key issues In: Klingemann H, Sobell LC, editors. Promoting Self-Change From Addictive Behaviors New York: Springer pp. 1–30.

[15] Chapman S, MacKenzie R (2010) The global research neglect of unassisted smoking cessation: causes and consequences. PLoS Medicine 7: e1000216 10.1371/journal.pmed.1000216 20161722 PMC2817714

[16] Kluge A (2009) A qualitative inquiry into smoking cessation: lessons learned from smokers. Graduate School of Emory University. Accessed 2 December 2013.

[17] Dixon-Woods M, Agarwal S, Young B, Jones D, Sutton A (2004) Integrative approaches to qualitative and quantitative evidence London: Health Development Agency.

[18] Atkins S, Lewin S, Smith H, Engel M, Fretheim A, Volmink J (2008) Conducting a meta-ethnography of qualitative literature: lessons learnt. BMC Med Res Methodol 8: 10.1186/1471-2288-8-21 PMC2374791 18416812

[19] Barnett-Page E, Thomas J (2009) Methods for the synthesis of qualitative research: a critical review. BMC Med Res Methodol 9: 10.1186/1471-2288-9-59 PMC3224695 19671152

[20] Thomas J, Harden A (2008) Methods for the thematic synthesis of qualitative research in systematic reviews. BMC Med Res Methodol 8: 10.1186/1471-2288-8-45 PMC2478656 18616818

[21] McDermott E, Graham H, Hamilton V (2004) Experiences of being a teenage mother in the UK: a report of a systematic review of qualitative studies Glasgow: The Social and Public Health Services Unit, University of Glasgow.

[22] Noyes J, Popay J, Pearson A, Hannes K, Booth A (2008) Cochrane handbook for systematic reviews of interventions Chichester: Wiley-Blackwell.

[23] Wong SS, Wilczynski NL, Haynes RB (2004) Developing optimal search strategies for detecting clinically relevant qualitative studies in MEDLINE. Medinfo 11: 311–316. 15360825

[24] Wilczynski NL, Marks S, Haynes RB (2007) Search strategies for identifying qualitative studies in CINAHL. Qual Health Res 17: 705–710. 17478652 10.1177/1049732306294515

[25] McKibbon KA, Wilczynski NL, Haynes RB (2006) Developing optimal search strategies for retrieving qualitative studies in PsycINFO. Evaluation & the Health Professions 29: 440–454. 17102065 10.1177/0163278706293400

[26] Sandelowski M, Barroso J (2007) Handbook for synthesizing qualitative research New York: Springer Publishing Company.

[27] Saini M, Shlonsky A (2012) Systematic synthesis of qualitative research Oxford: Oxford University Press.

[28] Baer PE, Foreyt JP, Wright S (1977) Self-directed termination of excessive cigarette use among untreated smokers. Journal of Behavior Therapy and Experimental Psychiatry 8: 71–74.

[29] Solheim K (1989) The smoking cessation process. Journal of Holistic Nursing 7: 26–33. 11898217

[30] Thompson EM (1995) A descriptive study of women who have successfully quit smoking. PhD Thesis, Georgia State University. Accessed 16 January 2014.

[31] Mariezcurrena R (1996) Recovery from addictions without treatment: an interview study. Cognitive Behaviour Therapy 25: 57–85.

[32] Stewart C (1999) Investigation of cigarette smokers who quit without treatment. Journal of Drug Issues 29: 167–186.

[33] Abdullah AS, Ho WW (2006) What Chinese adolescents think about quitting smoking: a qualitative study. Substance Use & Misuse 41: 1735–1743. 17118813 10.1080/10826080601006433

[34] Nichter M, Nichter M, Muramoto M, Adrian S, Goldade K, Tesler L, et al (2007) Smoking among low-income pregnant women: an ethnographic analysis. Health Education & Behavior 34: 748–764. 17200098 10.1177/1090198106290397

[35] Ogden J, Hills L (2008) Understanding sustained behavior change: The role of life crises and the process of reinvention. Health: An Interdisciplinary Journal for the Social Study of Health, Illness and Medicine 12: 419–437. 10.1177/1363459308094417 18818273

[36] Bottorff JL, Radsma J, Kelly M, Oliffe JL (2009) Fathers' narratives of reducing and quitting smoking. Sociology of Health & Illness 31: 185–200. 19055591 10.1111/j.1467-9566.2008.01126.x

[37] Murray RL, McNeill A, Lewis S, Britton J, Coleman T (2010) Unplanned attempts to quit smoking: a qualitative exploration. Addiction 105: 1299–1302. 10.1111/j.1360-0443.2010.02980.x 20642512

[38] Medbø A, Melbye H, Rudebeck CE (2011) "I did not intend to stop. I just could not stand cigarettes any more." A qualitative interview study of smoking cessation among the elderly. BMC Family Practice 12:42: 10.1186/1471-2296-12-42 21627833 PMC3132720

[39] Stewart JC (1998) A qualitative investigation of informants who quit cigarette smoking without treatment. PhD Thesis, Florida State University. Accessed 9 February 2014.

[40] Murray RL (2009) Investigating and increasing smokers' use of effective cessation support. PhD Thesis, University of Nottingham. Available: http://ethos.bl.uk/OrderDetails.do?did=1&uin=uk.bl.ethos.514878. Accessed 2013 Dec 5.

[41] Dixon-Woods M, Sutton A, Shaw R, Miller T, Smith J, Young B, et al (2007) Appraising qualitative research for inclusion in systematic reviews: a quantitative and qualitative comparison of three methods. J Health Serv Res Policy 12: 42–47. 17244397 10.1258/135581907779497486

[42] Eakin JM, Mykhalovskiy E (2003) Reframing the evaluation of qualitative health research: reflections on a review of appraisal guidelines in the health sciences. Journal of Evaluation in Clinical Practice 9: 187–194. 12787182 10.1046/j.1365-2753.2003.00392.x

[43] Lipworth WL, Hooker C, Carter SM (2011) Balance, balancing, and health. Qual Health Res 21: 714–725. 10.1177/1049732311399781 21343435

[44] Charmaz K (2006) Constructing grounded theory: a practical guide through qualitative analysis Thousand Oaks, California: Sage Publications.

[45] Hung WT, Dunlop SM, Perez D, Cotter T (2011) Use and perceived helpfulness of smoking cessation methods: results from a population survey of recent quitters. BMC Public Health 11: 592–600. 10.1186/1471-2458-11-592 21791111 PMC3160379

[46] GALLUP Poll Social Series: Consumption habits. Available http://www.gallup.com/poll/163763/smokers-quit-tried-multiple-times.aspx. Accessed 10 October 2013.

[47] Shaw RL, Booth A, Sutton AJ, Miller T, Smith JA, Young B, et al (2004) Finding qualitative research: an evaluation of search strategies. BMC Med Res Methodol 4: 5 15070427 10.1186/1471-2288-4-5 PMC385230

[48] Carey MP, Snel DL, Carey KB, Richards CS (1989) Self-initiated smoking cessation: a review of the empirical literature from a stress and coping perspective. Cognitive Therapy and Research 13: 323–341.

[49] Cohen S, Lichtenstein E, Prochaska JO, Rossi JS, Gritz ER, Carr CR, et al (1989) Debunking myths about self-quitting: evidence from 10 prospective studies of persons who attempt to quit smoking by themselves. American Psychologist 44: 1355–1365. 2589730 10.1037//0003-066x.44.11.1355

[50] Ockene JK, Mermelstein RJ, Bonollo DS, Emmons KM, Perkins KA, Voorhees CC, et al (2000) Relapse and maintenance issues for smoking cessation. Health Psychology 19: 17–31. 10709945 10.1037/0278-6133.19.suppl1.17

[51] Prochaska JO, DiClemente CC (1982) Self change processes, self efficacy and decisional balance across five stages of smoking cessation. Progress in Clinical and Biological Research 156: 131–140. 6473420

[52] DiClemente CC, Prochaska JO (1982) Self-change and therapy change of smoking behavior: a comparison of processes of change in cessation and maintenance. Addict Behav 7: 133–142. 7102444 10.1016/0306-4603(82)90038-7

[53] Borland R, Yong H-H, Balmford J, Cooper J, Cummings KM, O’Connor RJ, et al (2010) Motivational factors predict quit attempts but not maintenance of smoking cessation: findings from the International Tobacco Control Four Country project. Nicotine Tob Res 12 Suppl: S4–11. 10.1093/ntr/ntq050 20889479 PMC2948136

[54] Smit ES, Fidler JA, West R (2011) The role of desire, duty and intention in predicting attempts to quit smoking. Addiction 106: 844–851. 10.1111/j.1360-0443.2010.03317.x 21205048

[55] Nezami E, Sussman S, Pentz MA (2003) Motivation in tobacco use cessation research. Substance Use & Misuse 38: 25–50. 12602805 10.1081/ja-120016564

[56] Balmford J, Borland R, Hammond D, Cummings KM (2011) Adherence to and reasons for premature discontinuation from stop-smoking medications: data from the ITC Four-Country Survey. Nicotine Tob Res 13: 94–102. 10.1093/ntr/ntq215 21147894 PMC3028191

[57] Prochaska J, DiClemente C (1994) Stages of change in the modification of problem behaviors In: Hersen M, Eisler R, Miller P, editors. Progress in behavior modification Sycamore, IL: Academic Press pp. 184–214. 1620663

[58] Segan CJ, Borland R, Greenwood KM (2002) Do transtheoretical model measures predict the transition from preparation to action in smoking cessation? Psychol Health 17: 417–435.

[59] Kahler CW, Lachance HR, Strong DR, Ramsey SE, Monti PM, Brown RA (2007) The commitment to quitting smoking scale: initial validation in a smoking cessation trial for heavy social drinkers. Addict Behav 32: 2420–2424. 17478057 10.1016/j.addbeh.2007.04.002 PMC1986789

[60] Stoptober challenge reaches new high as country’s biggest mass quit attempt. Available https://www.gov.uk/government/news/stoptober-challenge-reaches-new-high-as-countrys-biggest-mass-quit-attempt Accessed 2014 Jun 26.

[61] Etter J-F, Bergman MM, Humair J-P, Perneger TV (2000) Development and validation of a scale measuring self-efficacy of current and former smokers. Addiction 95: 901–913. 10946439 10.1046/j.1360-0443.2000.9569017.x

[62] (2004) Handbook of self-regulation: research, theory, and applications New York: Guilford Press.

[63] Deci EL, Ryan RM (2010) Self-determination The Corsini Encyclopedia of Psychology Hoboken, NJ, USA: John Wiley & Sons, Inc. pp. 1–2.

[64] Orford J, Hodgson R, Copello A, John B, Smith M, Black R, et al (2006) The clients’ perspective on change during treatment for an alcohol problem: qualitative analysis of follow-up interviews in the UK Alcohol Treatment Trial. Addiction 101: 60–68. 16393192 10.1111/j.1360-0443.2005.01291.x

[65] McDermott E, Graham H (2006) Young mothers and smoking: evidence of an evidence gap. Social Science & Medicine 63: 1546–1549. 16730110 10.1016/j.socscimed.2006.03.025

